# Baseline assessment of patient safety culture in public hospitals in Kuwait

**DOI:** 10.1186/s12913-018-2960-x

**Published:** 2018-03-06

**Authors:** Hayfaa Ali, Samaa Zenhom Ibrahem, Buthaina Al Mudaf, Talal Al Fadalah, Diana Jamal, Fadi El-Jardali

**Affiliations:** 1 Ministry of Health, Kuwait City, Kuwait; 2 Department of Health Management, Planning and Policy, High Institute of Public Health, Alexandria University, Alexandria, Egypt; 3 Department of Health Management and Policy, Faculty of Heath Sciences, American University of Beirut, Beirut, Lebanon

**Keywords:** Kuwait, Public hospitals, Baseline assessment, Benchmark, Hospital survey on patient safety culture

## Abstract

**Background:**

Conducting patient safety culture assessments can provide hospitals with information on how structures and processes within their system can impact patient outcomes. This study used the Hospital Survey on Patient Safety Culture (HSOPSC) to conduct an assessment of patient safety culture in public hospitals in Kuwait and benchmark against regional and international studies that utilized the same tool. This objective of this study is to examine the association between the predictors and outcomes of patient safety culture.

**Methods:**

This cross sectional study adopted a customized version of HSOPSC developed by the Agency for Healthcare Research and Quality. The survey targeted selected public hospital staff with at least one year of experience. Data was analyzed using SPSS 24 at a significance level of 0.05. Univariate analysis was utilized to obtain an overview of respondent demographics. The association between patient safety grade and the number of events reported and the remaining patient safety culture composites was analyzed using ANOVA f-test. Four regression models were constructed, two adopted Generalized Estimating Equations and the others were linear models. Results were benchmarked against similar initiatives in Lebanon, Saudi Arabia and USA.

**Results:**

A total of 12,092 employees from 16 public hospitals in Kuwait completed the survey. The overall response rate was 60.5% (20,003 distributed surveys). Areas of strength were Teamwork within Units, Organizational Learning—Continuous Improvement, Management Support for Patient Safety, Supervisor/Manager Expectations & Actions Promoting Patient Safety, and Feedback and Communication about Error. Regression findings highlighted significant association between patient safety outcomes and composites. Benchmarking analysis revealed that Kuwaiti hospitals are performing at or better than benchmark on several composites compared to regional and international findings.

**Conclusion:**

This is the first major study addressing patient safety culture in public hospitals in Kuwait. Despite having some areas for improvement, public hospitals in Kuwait were found to have multiple areas of strength. Improving patient safety culture is critical if hospitals want to improve quality and safety of medical services. Study findings can guide and inform country level strategies to further improve the systems governing patient safety practices.

## Background

Patient safety culture reflect the values that members of the organization share regarding what is important, how things operate and how inter-departmental interactions, structures and systems are collectively manifested in behavioural norms that support patient safety [1]. It reflects a non-punitive organizational culture that encourages reporting, analysing and learning from medical errors [2]. Ever since the Institute of Medicine (IOM) recommended a patient safety culture for building safety into the processes of care [2], evidence has been accumulating on the importance of cultivating patient safety culture to reduce adverse events and improve patient safety.

Conducting an assessment of patient safety culture in hospitals is only the first step of defining and refining a solid safety culture [3]. Multiple international accreditation organizations have now require patient safety culture assessments within their standards so that hospitals can assess and evaluate issues such as teamwork, managerial actions, support from upper administration and leadership to support patient safety, staffing challenges, reporting of incidents, and other related issues [4]. This allows healthcare organizations to develop a clearer view of the areas where they need to focus their attention as part of their efforts to strengthen patient safety culture [5]. Furthermore, when hospitals conduct such assessments, they can also benchmark their results against similar initiatives conducted within their country or at an international level [6].

Before we embark onto developing and improving patient safety culture we must first diagnose its current state and patient safety culture surveys are pivotal to assess areas of strengths and weaknesses in patient safety culture. The most commonly used patient safety culture survey is the Hospital Survey on Patient Safety Culture (HSOPSC) which assesses patient safety culture based on 12 dimensions: Teamwork within Units, Supervisor/manager Expectations and Actions Promoting Patient Safety, Organizational Learning and Continuous Improvement, Management Support for Patient Safety, Overall Perceptions of Patient Safety, Feedback and Communication about Error, Communication Openness, Frequency of Events Reported, Teamwork across Units, Handoffs and Transitions, Staffing and Non punitive Response to Error [7]. The HSOPSC survey, which was developed by the Agency for Healthcare Research and Quality (AHRQ), has immense international reverberation as it has been validated and used in different continents and contexts [8].

Multiple studies and systematic reviews have tackled the issue of patient safety culture in the Arab world and beyond. A systematic review targeting Arab countries identified non-punitive response to error as a major challenge and healthcare professionals in these countries reported that culture of blame prevents them from reporting incidents [9]. Challenges pertaining to non-punitive response to error were also highlighted in Swedish hospitals [10], Tunisian operating rooms [11], and Iran [12]. Focusing on improving response to error is crucial to improving error reporting and in fact, the likelihood of voluntary incident reporting was found to improve by focusing efforts on cultural changes such as improving event feedback mechanisms and communication of event-related improvements [13]. Evidence has shown that feedback can positively stimulate improvement in patient safety culture if it is tailored to specific departments and if outcomes were comprehensible for intended users [14]. Other areas requiring improvement were also highlighted in related evidence. They included Teamwork across Units, Handoffs and Transitions, Staffing and Communication Openness [15].

Many areas of strength were also highlighted in the patient safety culture literature whereby a study in Iran found that organizational learning-continuous improvement, teamwork within hospital units, and hospital management support for patient safety were all areas of strength [12]. Moreover, teamwork within units was better than teamwork across hospital units in Arab countries [9]. When assessing findings in specific countries, areas of strength in Lebanese hospitals were mainly related to Teamwork within Units, Management Support for Patient Safety, and Organizational Learning and Continuous Improvement [15]. As for KSA, and specifically Riyadh, areas of strength related to Organizational Learning and Continuous Improvement and Teamwork within units [16].

Predictors of a strong and positive patient safety culture include communication, ensuring flow of information between and across units, sharing a common vision on patient safety, in addition to commitment from management and leadership, and a non-punitive outlook towards incident reporting [17]. Investing in patient safety culture and quality management systems has been highlighted in recent studies in the Arab world [11]. Improving patient safety culture may also indirectly improve consumer-focused publicly reported hospital rating scores [18].

Limited research was found in the context of Kuwait. One study focusing on patient safety culture in primary care settings identified non - punitive response to errors, frequency of event reporting, staffing, communication openness, and handoffs and transitions as areas of weakness. Areas of strength were identified as teamwork within units and organizational learning [19]. This study, however, used the hospital survey for primary care settings, a tool specific to medical offices is available on the AHRQ website. No other studies focusing on patient safety culture in Kuwait were identified.

### Objectives

The study aimed at assessing patient safety culture in public hospitals in Kuwait as perceived by hospital staff and compare results to those of similar regional and international studies. Furthermore, the study explored the association between patient safety culture predictors and outcomes, taking into consideration respondent characteristics.

## Methods

This cross sectional study utilized the Hospital Survey on Patient Safety Culture (HSOPSC) developed by the Agency for Healthcare Research and Quality. The survey has been customized to fit the context of Kuwait.

### Setting

The survey covered 16 public hospitals in Kuwait; two of the hospitals were small-sized (< 100 beds), another two were medium-sized (101–300 beds), and the remaining 12 hospitals were large (more than 300 beds). There are 20 public hospitals in Kuwait, however, we selected 16 hospitals as the remaining facilities had only recently been established and as such did not meet our inclusion criteria as detailed below.

### Sampling and data collection

The survey targeted selected hospital staff including physicians, nurses, pharmacy and laboratory staff, dietary and radiology staff, supervisors, and hospital managers. Data collection spanned 8 months (April to November 2015). Healthcare providers in the below mentioned categories were included in the study:

- Healthcare organization leaders (Hospital Director, Deputy Director, Assistant director).
- Heads of administrative departments (Social Services, Public Relations, Medical Records, Hotel Services, Accounting Services, Engineering, inventory, etc.).
- Physicians of all specialties and ranks (including those working in radiology, laboratory, nuclear medicine, etc.)
- Pharmacists
- Nurses
- Dieticians
- Infection control physicians and nurses including sterilization department staff.
- Quality physicians and nurses
- Physical, occupational, and speech therapist/ technicians
- Technicians (sterilization, lab, radiology, anaesthesia, pharmacy etc.)
- Medical engineering staff
- Medical records staff

Exclusion criteria:

- Staff on administrative or extended sick leave,
- Staff who have moved to another hospital area/unit, and
- Staff with less than 1 year of experience in the hospital.

Surveys were distributed through an assigned focal person at every hospital. A pre-determined number of surveys were sent to each hospital based on the total number of eligible employees. The surveys were coded with two numbers, one representing the hospital and the other representing the survey. Focal people were asked not to make copies of the survey so as not to jeopardize the integrity of the coding scheme determined by the research team. Surveys were distributed during department meetings or via departmental secretaries. Respondents were asked to refrain from writing their names or any information that would identify them on any page of the survey but they were asked to sign the consent form to verify that they read the information provided in it. They were asked to complete the survey and enclose it in a provided envelope, seal the envelope and return to a confidential drop box within each department.

### Study instrument

The HSOPSC survey was utilized. The tool was translated to Arabic to account for employees who are not very comfortable with English. The Arabic version of the survey was adapted from El-Jardali et al. [15, 20].

Pilot testing was conducted with 20 employees who did not participate in the consequent phases of the study. Minor changes were made to the wording and categories within some questions as a result of piloting.

### Ethical approval

Ethical clearance to conduct the survey was provided by the Standing Committee for Coordination of Health and Medical Research in Kuwait.

### Data management and analysis

Data was analyzed using SPSS 24.0 (*p*-value = 0.05). The survey tool includes 42 items which measure 12 composites. The items are both positively and negatively wordedwhich are scored using a five-point scale reflecting respondent agreement/frequency (including a neutral category). Percent positive response within each composite was calculated. Negatively worded items were reversed prior to calculation of percent positive per composite. The full calculation method has been mentioned in El-Jardali et al. [15, 20] Internal consistency was calculated using Cronbach’s alpha.

The survey has a total of 4 outcome variables. The first two are frequency of events reported and overall perception of safety which are measured within the 12 composites [7]. The remaining two outcome variables are the patient safety grade and the number of events reported which are measured as separate multiple choice questions [7].

Bi-variate analysis included ***ANOVA*** f-test was used to examine the association between the two outcome variables with the patient safety culture composites. Student ***T-Test*** and ***ANOVA*** f-test were then used to examine how trends in the outcome variables differ across hospital and respondent characteristics.

The four outcome variables were regressed against the 10 composite scores, respondent and hospital characteristics. Four regression models were constructed, two adopted ***Generalized Estimating Equations*** (the two categorical outcome variables: number of events reported and patient safety grade) and the other two models followed a ***Linear Mixed Regression Model*** (the two composites for frequency of events reported and overall perception of safety). In the latter models, the independent variables were entered as dummy variables. The two categorical outcomes were recoded into fewer categories for the purpose of this analysis. The outcome on patient safety grade was recoded into three categories “Poor or Failing,” “Acceptable,” and “Excellent/Good.” The outcome on number of events reported was recoded into “> 5 events reported,” “1 to 5 events reported,” and “No events reported.”

Results from the 16 participating hospitals were also benchmarked against similar initiatives in the United States (US) [21] and Lebanon [20]. Comparison to the benchmark value was done using the below formula [7]:

%Distance from benchmark = ((benchmark value – hospital result)/benchmark value) ∗ 100

Values below 10% were categorized as meeting or exceeding benchmark. Those between 10 and 50% were categorized as slightly deviating from benchmark. Those exceeding 50% were categorized as highly deviating from benchmark.

## Results

A total of 12,871 employees from 16 public hospitals in Kuwait completed the patient safety culture survey. However, some hospitals sampled respondents with less than 1 year of experience, and as such these 779 responses were removed from the dataset giving a total of 12,092 surveys. The overall response rate based on the final 12,092 surveys was 60.5% (20,003 distributed surveys).

### Demographics

The majority of the sampled respondents were female (71.4%) and most were found to hold a university level degree (72.3%). Most of the sampled respondents were found to be nurses (66.8%), while 11.9% were physicians and 11.5% technicians. The majority of respondents (91.6%) indicated having patient interaction. Moreover, 86.4% were non-Kuwaiti. Finally, the majority of respondents worked in large hospitals (94.4%) while 3.2% worked in small hospitals and 2.4% worked in medium hospitals (See Table 1).

**Table 1:** Demographic characteristics of sample

	Number	Percent
Gender		
Male	3406	28.6
Female	8508	71.4
Education		
High school or below	572	4.8
University	8551	72.3
Technical	2573	21.7
Other	137	1.2
Profession		
Physician	1425	11.9
Pharmacist	283	2.4
Nurse	7987	66.8
Physiotherapist	434	3.6
Technician	1381	11.5
Nutritionist/Dietician	84	0.7
Administration	121	1
Medical Records	191	1.6
Others	56	0.5
Experience in Hospital		
Physician	1425	11.9
Pharmacist	283	2.4
Nurse	7987	66.8
Physiotherapist	434	3.6
Technician	1381	11.5
Nutritionist/Dietician	84	0.7
Administration	121	1
Medical Records	191	1.6
Others	56	0.5
Interaction with patients		
Yes	10,838	91.6
No	993	8.4
Nationality		
Kuwaiti	1609	13.6
Non-Kuwaiti	10,255	86.4

### Areas of strengths and areas requiring improvement

The twelve dimensions were examined to determine areas of strength (those with a positive rating of 70% or higher) and those requiring improvement (scoring below 70%) [22].

The dimensions with the highest positive score and are thus considered areas of strength were Teamwork within Units (89.7%), Organizational Learning—Continuous Improvement (86.1%), Management Support for Patient Safety (77.8%), Supervisor/Manager Expectations & Actions Promoting Patient Safety (77.1%), and Feedback and Communication about Error (70.7%). The remaining seven dimensions can be considered areas requiring improvement, they are teamwork across units (64.1%), handoffs and transitions (62.2%), overall perception of patient safety (60.6%), frequency of events reported (59.0%), communication openness (46.9%), staffing (39.9%) and non-punitive response to error (27.7%) (Table 2).

**Table 2:** Percent positive per item and per subscale^*^

	% Positive	% Neutral	% Negative
1. Teamwork Within Units			
People support one another in this unit. (A1)	94.9	2.9	2.2
When a lot of work needs to be done quickly, we work together as a team to get the work done. (A3)	93.1	4.1	2.9
In this unit, people treat each other with respect. (A4)	90.9	6.1	3.1
When one area in this unit gets really busy, others help out. (A11)	79.9	8.8	11.3
Average Teamwork Within Units	89.7	5.5	4.9
2. Supervisor/Manager Expectations & Actions Promoting Patient Safety			
My supervisor/manager says a good word when he/she sees a job done according to established patient safety procedures. (B1)	80.4	11.3	8.2
My supervisor/manager seriously considers staff suggestions for improving patient safety. (B2)	83.9	10.0	6.1
Whenever pressure builds up, my supervisor/manager wants us to work faster, even if it means taking shortcuts. (B3R)	61.3	17.5	21.2
My supervisor/manager overlooks patient safety problems that happen over and over. (B4R)	82.6	8.0	9.4
Average Supervisor/Manager Expectations & Actions Promoting Patient Safety	77.1	11.7	11.2
3. Organizational Learning—Continuous Improvement			
We are actively doing things to improve patient safety. (A6)	95.1	3.1	1.8
Mistakes have led to positive changes here. (A9)	76.0	14.1	9.9
After we make changes to improve patient safety, we evaluate their effectiveness. (A13)	87.2	8.0	4.7
Average Organizational Learning—Continuous Improvement	86.1	8.4	5.5
4. Management Support for Patient Safety			
Hospital management provides a work climate that promotes patient safety. (F1)	81.3	10.4	8.3
The actions of hospital management show that patient safety is a top priority. (F8)	86.1	8.5	5.4
Hospital management seems interested in patient safety only after an adverse event happens. (F9R)	65.9	13.7	20.4
Average Management Support for Patient Safety	77.8	10.9	11.4
5. Overall Perceptions of Patient Safety			
It is just by chance that more serious mistakes don’t happen around here. (A10R)	36.2	15.1	48.6
Patient safety is never sacrificed to get more work done. (A15)	79.7	6.1	14.3
We have patient safety problems in this unit. (A17R)	45.2	15.6	39.2
Our procedures and systems are good at preventing errors from happening. (A18)	81.1	10.6	8.2
Average Overall Perceptions of Patient Safety	60.6	11.9	27.6
6. Feedback and Communication About Error			
We are given feedback about changes put into place based on event reports. (C1)	50.8	29.6	19.6
We are informed about errors that happen in this unit. (C3)	79.9	14.1	6.1
In this unit, we discuss ways to prevent errors from happening again. (C5)	81.5	12.7	5.8
Average Feedback and Communication About Error	70.7	18.8	10.5
7. Communication Openness			
Staff will freely speak up if they see something that may negatively affect patient care. (C2)	67.7	20.7	11.6
Staff feel free to question the decisions or actions of those with more authority. (C4)	30.0	28.3	41.7
Staff are afraid to ask questions when something does not seem right. (C6R)	43.1	36.7	20.2
Average Communication Openness	46.9	28.6	24.5
8. Frequency of Events Reported			
When a mistake is made, but is caught and corrected before affecting the patient, how often is this reported? (D1)	55.5	20.4	24.1
When a mistake is made, but has no potential to harm the patient, how often is this reported? (D2)	54.7	21.7	23.6
When a mistake is made that could harm the patient, but does not, how often is this reported? (D3)	66.9	14.3	18.8
Average Frequency of Events Reported	59.0	18.8	22.2
9. Teamwork Across Units			
Hospital units do not coordinate well with each other. (F2R)	55.9	16.5	27.7
There is good cooperation among hospital units that need to work together. (F4)	71.1	15.6	13.3
It is often unpleasant to work with staff from other hospital units. (F6R)	46.3	21.1	32.6
Hospital units work well together to provide the best care for patients. (F10)	82.9	10.7	6.4
Average Teamwork Across Units	64.1	16.0	20.0
10. Staffing			
We have enough staff to handle the workload. (A2)	60.8	11.9	27.3
Staff in this unit work longer hours than is best for patient care. (A5R)	27.6	16.7	55.7
We use more agency/temporary staff than is best for patient care. (A7R)	52.5	19.5	27.9
We work in “crisis mode” trying to do too much, too quickly. (A14R)	18.5	13.8	67.7
Average Staffing	39.9	15.5	44.7
11. Handoffs & Transitions			
Things “fall between the cracks” when transferring patients from one unit to another. (F3R)	54.6	18.7	26.7
Important patient care information is often lost during shift changes. (F5R)	75.5	12.5	12.1
Problems often occur in the exchange of information across hospital units. (F7R)	48.5	24.2	27.3
Shift changes are problematic for patients in this hospital. (F11R)	70.3	15.5	14.2
Average Handoffs & Transitions	62.2	17.7	20.1
12. Non-punitive Response to Error			
Staff feel like their mistakes are held against them. (A8R)	29.5	19.5	50.9
When an event is reported, it feels like the person is being written up, not the problem. (A12R)	38.1	18.4	43.4
Staff worry that mistakes they make are kept in their personnel file. (A16R)	15.6	13.7	70.8
Average Non-punitive Response to Error	27.7	17.2	55.0

^*^the composite-level percentage of positive responses was calculated using the following formula: (number of positive responses to the items in the composite/total number of responses to the items (positive, neutral, and negative) in the composite (excluding missing responses))*100

(R) Negatively worded items that were reverse coded

Items considered areas of strength and others which require improvement were examined. The biggest area of strength highlighted by the responses was the item related to the hospital taking action to improve patient safety to which percent positive response was 95.1%. Other areas of strength were revealed within the dimension on Teamwork within units whereby the item on whether staff support one another within a unit received 94.9% positive responses, working together as a team when a lot of work needs to be done quickly (93.1% positive) and treating each other with respect within the unit (90.9% positive) (See Table 2).

The area with the lowest percent positive related to the dimension on non-punitive response to error whereby staff worry that their mistakes are kept in their personnel files (15.6% positive, reverse item). Another item within this dimension that was found to be an area requiring improvement related to staff feeling their mistakes are held against them (29.5% positive, reverse item). The dimension on staffing also emerged as problematic as staff indicated trying to do too much too quickly when working in crisis mode (18.5% positive, reverse item). Moreover, 27.6% of responses indicate that staff work longer hours than is best for patient safety (reverse item). The dimension relating to communication openness was also found to be an area requiring improvement where only 30.0% of the staff feel free to question the decisions or actions of those with more authority and 43.1% only are not afraid to ask questions when something does not seem right (reverse item).

Results on areas of strength and areas requiring improvement are fully detailed in Table 2.

### Association between patient safety grade and number of events with composites

Respondents who gave “Excellent/Very Good” patient safety grades had significantly the highest mean scores for patient safety composites (See Table 3). Teamwork within Hospital Units and Organizational Learning-Continuous Improvement demonstrated the highest mean score in relation to patient safety grade, while the Non-punitive Response to Error and staffing scored the lowest in relation to patient safety grade. The number of events reported was significantly associated with all of the patient safety composites. The highest mean observed when reporting more than 5 events was for the composite measuring Teamwork within Hospital Units while the lowest was observed for Non-punitive Response to Error (Table 3).

**Table 3:** Comparison between patient safety grade and number of events reported with patient safety culture composite scores (composites scored range from 1 to 5)

	Patient Safety Grade				Number of Events Reported			
	Sig.	Poor or Failing	Acceptable	Excellent/ Very Good	Sig.	No event reports	1 to 5 event reports	> 5 events reported
		Mean (SD)	Mean (SD)	Mean (SD)		Mean (SD)	Mean (SD)	Mean (SD)
Supervisor/manager expectations and actions promoting safety	a,b,c	3.15 (0.88)	3.62 (0.62)	3.94 (0.56)		3.87 (0.60)	3.85 (0.60)	3.86 (0.61)
Organizational Learning-Continuous Improvement	a,b,c	3.39 (0.89)	3.85 (0.61)	4.16 (0.47)	a,b	4.07 (0.54)	4.10 (0.52)	4.13 (0.55)
Teamwork Within Hospital Units	a,b,c	3.64 (0.92)	3.96 (0.62)	4.26 (0.49)		4.19 (0.56)	4.19 (0.52)	4.19 (0.58)
Communication Openness	a,b,c	2.59 (0.94)	3.01 (0.83)	3.46 (0.80)	a	3.40 (0.83)	3.31 (0.83)	3.35 (0.86)
Feedback and Communication About Errors	a,b,c	2.87 (1.08)	3.60 (0.83)	4.10 (0.69)	a,c	3.98 (0.78)	3.97 (0.75)	4.03 (0.76)
Non-punitive Response to Error	b,c	2.38 (0.84)	2.44 (0.73)	2.69 (0.74)	a,b	2.66 (0.74)	2.60 (0.76)	2.60 (0.76)
Staffing	b,c	2.72 (0.62)	2.80 (0.55)	2.93 (0.55)	a,b	2.92 (0.56)	2.87 (0.54)	2.84 (0.57)
Hospital Management Support for Patient Safety	a,b,c	2.68 (0.93)	3.44 (0.69)	3.96 (0.61)	a,b	3.88 (0.69)	3.80 (0.67)	3.74 (0.78)
Hospital Handoffs and Transitions	a,b,c	2.92 (0.88)	3.23 (0.75)	3.56 (0.71)	a,b	3.51 (0.71)	3.47 (0.74)	3.36 (0.80)
Teamwork Across Hospital Units	a,b,c	2.69 (0.85)	3.20 (0.67)	3.63 (0.63)	a,b,c	3.56 (0.67)	3.50 (0.67)	3.44 (0.72)
Patient Safety Grade a. Significant difference between “Poor or Failing” and “Acceptable” b. Significant difference between “Poor or Failing” and “Excellent/Very Good” c. Significant difference between “Acceptable” and “Excellent/Very Good”				Number of Events Reported a. Significant difference between “No events reported” and “1 to 5 events reported” b. Significant difference between “No events reported” and “> 5 events reported” c. Significant difference between “1 to 5 events reported” and “> 5 events reported”				

### Generalized estimating equations

As detailed below, a one unit increase on composites relating to Supervisor/Manager Expectations & Actions Promoting Patient Safety, Organizational learning and Continuous Improvement, Teamwork within units, Communication Openness, Feedback and Communications about Error, Staffing, Hospital Management Support for Patient Safety, and Teamwork across Hospital Units resulted in higher odds of reporting better patient safety grades. Similarly, a one unit increase on composites relating to Organizational learning and Continuous Improvement and Feedback and Communications about Error resulted in greater odds of reporting higher number of events while a one unit increase on Staffing and Hospital Management Support for Patient Safety resulted in lower odds of reporting number of events (Table 4).

**Table 4:** Generalized Estimating Equations

	Patient Safety Grade		Number of Events Reported	
	OR (95% CI)	*P*-value	OR (95% CI)	*P*-value
Patient Safety Culture Composites				
Supervisor/Manager Expectations & Actions Promoting Patient Safety	0.73 (0.67–0.80)	< 0.001	1.05 (0.95–1.16)	0.318
Organizational learning and Continuous Improvement	0.65 (0.55–0.77)	< 0.001	1.27 (1.16–1.39)	< 0.001
Teamwork within units	0.75 (0.68–0.83)	< 0.001	1.05 (0.95–1.15)	0.347
Communication Openness	0.78 (0.67–0.91)	0.002	0.91 (0.83–1.01)	0.077
Feedback and Communications About Error	0.72 (0.63–0.83)	< 0.001	1.10 (1.02–1.19)	0.018
Non-punitive Response to Error	1.01 (0.89–1.15)	0.850	0.96 (0.86–1.07)	0.448
Staffing	0.84 (0.72–0.97)	0.021	0.88 (0.78–0.99)	0.038
Hospital Management Support for Patient Safety	0.47 (0.40–0.54)	< 0.001	0.81 (0.71–0.92)	0.002
Hospital Handoffs & Transitions	0.89 (0.75–1.06)	0.197	0.94 (0.86–1.02)	0.137
Teamwork Across Hospital Units	0.81 (0.67–0.98)	0.027	0.94 (0.82–1.07)	0.318
Gender				
Male	1.11 (0.90–1.35)	0.300	1.14 (0.97–1.35)	0.122
Female	1		1	
Experience at the hospital				
< 5 years	0.87 (0.69–1.10)	0.237	1.12 (0.98–1.27)	0.086
5 to 20 years	0.90 (0.74–1.10)	0.289	0.98 (0.83–1.15)	0.765
More or equal to 21 years	1		1	
Highest Degree				
High school or below	1.19 (0.71–2.02)	0.505	1.21 (0.74–1.98)	0.437
University Degree	0.59 (0.35–1.00)	0.048	0.81 (0.55–1.19)	0.275
Technical Degree	0.89 (0.58–1.35)	0.577	0.95 (0.64–1.41)	0.795
Other	1		1	
Position at the hospital				
Physician	1.15 (0.86–1.53)	0.344	0.57 (0.47–0.69)	< 0.001
Pharmacist	1.12 (0.69–1.83)	0.637	0.52 (0.32–0.82)	0.005
Nurse	0.97 (0.73–1.29)	0.829	0.51 (0.43–0.59)	< 0.001
Admin	1.07 (0.61–1.87)	0.828	0.24 (0.14–0.43)	< 0.001
Other	1		1	
Nationality				
Kuwaiti	0.68 (0.49–0.93)	0.016	1.20 (1.04–1.37)	0.010
Non-Kuwaiti	1		1	
Interaction with patients				
Yes	1.01 (0.76–1.32)	0.967	0.82 (0.68–0.98)	0.033
No	1		1	
Hospital Size				
Small	1.65 (1.17–2.33)	0.004	2.67 (2.17–3.30)	< 0.001
Medium	2.02 (0.85–4.79)	0.110	1.85 (1.07–3.20)	0.028
Large	1		1	

Respondents holding a university degree were less likely to report better patient safety grades than those holding “other” degrees. Physicians, pharmacists, nurses and administrative staff, all had lower odds of reporting higher number of events compared to “other staff”. Kuwaiti nationals had lower odds of reporting better patient safety grade but higher odds of reporting higher number of events compared to non-nationals. Respondents who had contact with patients had lower odds of reporting higher number of events compared to respondents with no patient contact. Respondents were more likely to report better patient safety grade as hospital size increased from small to medium. The opposite was observed for number of events where odds of reporting higher number of events decreased with increasing hospital size (Table 4).

### Linear mixed model regression

The Linear regression analysis in Table 5 showed that a one unit increase in the scores on Organizational learning and Continuous Improvement, Feedback and Communications about Error, Hospital Management Support for Patient Safety and Hospital Handoffs & Transitions resulted in higher frequency of events reporting. Similarly, a one unit increase in the scores on Supervisor/ Manager Expectations & Actions Promoting Patient Safety, Organizational learning and Continuous Improvement, Teamwork within units, Communication Openness, Non-punitive Response to Error, Staffing, Hospital Management Support for Patient Safety and Hospital Handoffs & Transitions resulted in a higher perceived patient safety. However, a one unit increase on Teamwork across Hospital Units resulted in a lower perceived patient safety (Table 5).

**Table 5:** Linear Mixed Model Regression

	Frequency of Events Reported		Perception of Patient Safety	
	Beta (Standard Error)	*P*-value	Beta (Standard Error)	*P*-value
Patient Safety Culture Composites				
Supervisor/ Manager Expectations & Actions Promoting Patient Safety	0.03 (0.02)	0.112	0.12 (0.01)	< 0.001
Organizational learning and Continuous Improvement	0.09 (0.02)	< 0.001	0.13 (0.01)	< 0.001
Teamwork within units	0.01 (0.02)	0.796	0.10 (0.01)	< 0.001
Communication Openness	0.02 (0.01)	0.249	0.02 (0.01)	0.002
Feedback and Communications About Error	0.32 (0.02)	< 0.001	−0.01 (0.01)	0.322
Non-punitive Response to Error	0.02 (0.01)	0.150	0.03 (0.01)	< 0.001
Staffing	−0.01 (0.02)	0.457	0.11 (0.01)	< 0.001
Hospital Management Support for Patient Safety	0.07 (0.02)	< 0.001	0.12 (0.01)	< 0.001
Hospital Handoffs & Transitions	0.07 (0.02)	< 0.001	0.09 (0.01)	< 0.001
Teamwork Across Hospital Units	−0.02 (0.02)	0.200	−0.04 (0.01)	< 0.001
Gender				
Male	−0.08 (0.02)	< 0.001	0.05 (0.01)	< 0.001
Female	0		0	
Highest Degree				
High School or below	0.05 (0.10)	0.598	0.16 (0.05)	0.001
University or Higher Degree	−0.17 (0.09)	0.046	0.16 (0.04)	< 0.001
Technical Degree	−0.10 (0.09)	0.283	0.18 (0.04)	< 0.001
Other	0		0	
Experience at the hospital				
< 5 years	0.05 (0.03)	0.112	0.01 (0.02)	0.680
5 to 20 years	0.01 (0.03)	0.825	−0.01 (0.02)	0.422
More or equal to 21 years	0		0	
Profession				
Physician	−0.26 (0.14)	0.064	−0.11 (0.07)	0.116
Pharmacist	−0.25 (0.15)	0.097	0.00 (0.08)	0.996
Nurse	−0.44 (0.14)	0.002	0.13 (0.07)	0.059
Physiotherapist	−0.18 (0.15)	0.220	−0.08 (0.07)	0.292
Technician	−0.22 (0.14)	0.125	−0.05 (0.07)	0.484
Nutritionist	−0.18 (0.18)	0.305	0.09 (0.09)	0.314
Administrative	−0.30 (0.17)	0.073	0.09 (0.08)	0.255
Medical Records	−0.48 (0.16)	0.002	0.03 (0.08)	0.679
Other	0		0	
Nationality				
Kuwaiti	−0.09 (0.03)	0.005	0.08 (0.02)	< 0.001
Non-Kuwaiti	0		0	
Interaction with patients				
Yes	0.07 (0.04)	0.052	0.02 (0.02)	0.386
No	0		0	
Hospital Size				
Small	0.14 (0.08)	0.127	0.05 (20.73)	0.664
Medium	0.05 (0.09)	0.593	0.05 (24.09)	0.182
Large	0		0	

Male respondents were more likely to report lower frequency of events but more like to report a higher perceived patient safety. Respondents holding university degrees were more likely to report higher frequency of events. As for perception of patient safety, respondents holding university and technical degree were both more likely to report better perception. Nurses and Medical Records staff were more likely to report a lower frequency of events. Kuwaiti nationals were less like to report higher number of events but more likely to report a higher perceived patient safety grade (Table 5).

### Benchmarking

In Table 6, the results are compared to the US benchmark. This benchmark was selected since it is the most recent (2013–2014) and reflects the results of a nation-wide survey. Results are also compared to national surveys from Lebanon [15] and KSA [16]. Kuwait results were at or better than US benchmark for the seven composites: Teamwork within units, Supervisor/manager expectations and actions promoting patient safety, Organizational learning-continuous improvement, Management Support for Patient Safety, Overall perception of patient safety, Feedback and communication about error, Teamwork across hospital units, and Hospital handoffs and transitions. Kuwait had composites scores that were at or better than benchmark for Lebanon for eight of the composites: Teamwork within units, Supervisor/manager expectations and actions promoting patient safety, Organizational learning-continuous improvement, Management Support for Patient Safety, Feedback and communication about error, Teamwork across hospital units, Staffing and Hospital handoffs and transitions. When compared with KSA, Kuwait results were at or better than benchmark for nine of the composites: Teamwork within units, Supervisor/manager expectations and actions promoting patient safety, Organizational learning-continuous improvement, Management Support for Patient Safety, Overall perception of patient safety, Feedback and communication about error, Communication openness, Frequency of events reported, Teamwork across hospital units, Staffing, Hospital handoffs and transitions, and Non-punitive response to error. Five dimensions deviated slightly from benchmark when comparing results to the US. When comparing results to Lebanon, four composites differed slightly from the benchmark and three when comparing results to KSA. However, none of the composites were found to be worse than US, Lebanon, or KSA (Table 6).

**Table 6:** Benchmarking Percent Positive on Survey Composites from Kuwait against those in US, Lebanon and KSA

Composite	Kuwait	Benchmark US		Benchmark Lebanon		Benchmark KSA	
Teamwork within units	89.7%	81%	☑	82.3%	☑	78.50%	▣
Supervisor/manager expectations and actions promoting patient safety	77.0%	76%	☑	66.4%	▣	60.60%	▣
Organizational learning-continuous improvement	86.1%	73%	▣	78.3%	☑	79.60%	☑
Management Support for Patient Safety	77.7%	72%	☑	78.4%	☑	71.40%	☑
Overall perception of patient safety	60.5%	66%	☑	72.5%	▣	58.20%	☑
Feedback and communication about error	70.6%	67%	☑	68.1%	☑	63.30%	☑
Communication openness	47.2%	62%	▣	57.3%	▣	42.90%	☑
Frequency of events reported	58.8%	66%	☑	68.2%	☑	59.40%	☑
Teamwork across hospital units	63.8%	61%	☑	56.0%	☑	61.60%	☑
Staffing	39.6%	55%	▣	36.8%	☑	35.10%	☑
Hospital handoffs and transitions	61.9%	47%	▣	49.7%	▣	51.50%	▣
Non-punitive response to error	27.6%	44%	▣	24.3%	☑	26.80%	☑

☑Meets or better than benchmark (results within 10% of benchmark)

▣Deviates slightly from benchmark (results 10–50% from benchmark)

☒ Deviation from benchmark (results exceeding 50% difference with benchmark)

## Discussion

This is the first major study addressing patient safety culture in public hospitals in Kuwait. Despite having some areas for improvement, public hospitals in Kuwait were found to have multiple areas of strength especially with unit-level dimensions. Some critical unit-level dimensions such as staffing, communication openness, and non-punitive response to error are highly determined by overall hospital culture and systems that enable action within these dimensions. Hospital management should work hard on addressing these issues to improve reporting, overall perception of patient safety and patient safety grade.

The composite on non-punitive response scored lowest which is consistent with findings in the region and across the world. This reflects a need to invest in system improvement initiatives and strengthen patient safety culture when trying to addressing medical errors. Hospitals that have poorly developed and ineffective policies cannot prevent errors and as a result, cannot improve reporting and ultimately impact patient safety [23]. Fear of punishment has been consistently found to reduce frequency of error reporting [24] and this is confirmed in regression findings.

The finding linking better events reporting with the composite on Management Support of Patient Safety supports evidence that links supervisory communication to improved patient safety culture. Engaging staff, discussing quality challenges, and collectively developing solutions gives employees ownership and pride in improving patient safety [25]. Findings clearly demonstrate the need to encourage health professionals to report more events given their impact in improving patient safety. The three major components of a positive patient safety culture are: a just culture, a reporting culture, and a learning culture [26]. Better reporting is highly dependent on having a non-punitive environment where employees do not fear reporting events [5]. A punitive work environment is not a strange concept to hospitals in the region as it was reported to be an area for improvement in Lebanon and KSA [15, 16].

The association between hospital size and patient safety culture outcomes is also of note. In particular, medium-sized and small-sized hospitals were found to have better reporting of events and better patient safety grade. This is consistent with research that states that large hospitals face challenges in the implementation of quality improvement initiatives because of bureaucracy while smaller hospitals with a more homogenous culture are more likely to have staff members who share similar values [27].

Findings in this study showed that nurses are likely to report less events. This is critical as evidence in the literature indicate that nurses intercept 86% of potential errors [28]. Moreover, errors often go underreported for a multitude of reasons such as fear, humiliation, a punitive culture of reporting, or limited follow up after reporting an error [29].

Regression results indicate that employees who reported interaction with patients had fewer number of events and lower frequency of events reported. This is contrary to evidence in the literature which indicates that employees who have less interaction with patients are more at ease when reporting errors [30].

Benchmarking revealed many areas where Kuwaiti hospitals are performing at or better than benchmark and other areas of slight deviation. No major deviation from utilized benchmarks were observed. Comparing country findings to regional and international results can help hospital sets improvement goals and visualize their performance in comparison to others.

The main strength of this article lies in using a widely used and validated tool for assessing the culture of safety in hospitals at a national level. This study also utilized the Arabic version of the survey which was translated and validated in other Arab countries [15, 16, 20]. One limitation that should be highlighted is that nurses make up the majority of the sampled respondents. However, nurses comprise the majority of healthcare providers in most countries [31]. Despite having low representation from physicians, we were able to obtain input from a wide range of healthcare providers which can give a more comprehensive view on patient safety culture. Finally, the majority of respondents were non-Kuwaitis. However, that reflects the demographic distribution of the country and not only hospitals.

## Conclusion

This is the first large scale study that assesses patient safety culture in public hospitals in Kuwait. Improving patient safety culture is a critical if hospitals want to improve quality and safety of medical services. The overall culture within a hospital can reflect on the actions of hospitals with regard to safety and this can be revealed in patient outcomes. Study findings can guide and inform country level strategies to further improve the systems governing patient safety practices. Comparing findings to performance of other countries in the region can help hospitals and leaders visualize performance and set realistic targets for improvement. Investing in areas that affect overall patient safety culture, particularly event reporting, should be done if tangible improvement is to be made.

## Abbreviations

CI: Confidence interval
HSOPSC: Hospital survey on patient safety culture
KSA: Kingdom of Saudi Arabia
OR: Odds ratio
PSC: Patient safety culture
SD: Standard deviation
US: United States
